# Statistical exploration of dataset examining key indicators influencing housing and urban infrastructure investments in megacities

**DOI:** 10.1016/j.dib.2018.04.089

**Published:** 2018-05-02

**Authors:** Adedeji O. Afolabi, Rapheal A. Ojelabi, Adewale Bukola, Adedotun Akinola, Adesola Afolabi

**Affiliations:** a Department of Building Technology, Covenant University, Nigeria; b Department of Architecture, Covenant University, Nigeria; c Department of Economics and Development Studies, Covenant University, Nigeria

**Keywords:** Construction, Housing, Megacities, Population, Urban infrastructures

## Abstract

Lagos, by the UN standards, has attained the megacity status, with the attendant challenges of living up to that titanic position; regrettably it struggles with its present stock of housing and infrastructural facilities to match its new status. Based on a survey of construction professionals’ perception residing within the state, a questionnaire instrument was used to gather the dataset. The statistical exploration contains dataset on the state of housing and urban infrastructural deficit, key indicators spurring the investment by government to upturn the deficit and improvement mechanisms to tackle the infrastructural dearth. Descriptive statistics and inferential statistics were used to present the dataset. The dataset when analyzed can be useful for policy makers, local and international governments, world funding bodies, researchers and infrastructural investors.

**Specifications Table**

**Table:** 

Subject area	*Environmental Science*
More specific subject area	*Housing and urban development*
Type of data	*Tables and Figures*
How data was acquired	*Field Survey*
Data format	*Raw and analyzed*
Experimental factors	*Cross-sectional survey research design of architects, builders, urban planners, civil engineers and surveyors involved in housing and urban development projects*
Experimental features	*Sample selection, simple boxplot, stacked bars, correlation matrix and analysis of variance (ANOVA)*
Data source location	*Lagos, Nigeria*
Data accessibility	*All the data are in this data article*

**Value of the data**

• In many developing nations, there have been a rapid growth of urban population far more than that of rural population leading to tremendous expansion of urban areas and strain on the available infrastructure, therefore, the need for creating sustainable solutions.
• The success of administering and managing the affairs of megacities is hinged on adequate provision of quality and the right number of housing and urban infrastructure.
• Provision of housing and urban infrastructural facilities in developing economies are a herculean task that require the right strategies to fund them and bring them into existence. The dataset provides avenue for improving the infrastructural deficit that exist in megacities.
• Every economy needs to research into key indicators that would spur investment in housing and urban development projects due to the explosive urban growth the world is experiencing.
• The dataset can be replicated in other megacities, to understand the key infrastructures that need improvement to meet the needs of its citizenry and in order to cope with the rising population, there is need to factor the crucial indicators in planning for future housing and urban development projects.
• The dataset when analyzed can give insight into infrastructures that need a face-lift and reduce the rise of slums and blight areas within megacities.

## 1 Data

**Fig. 1:**
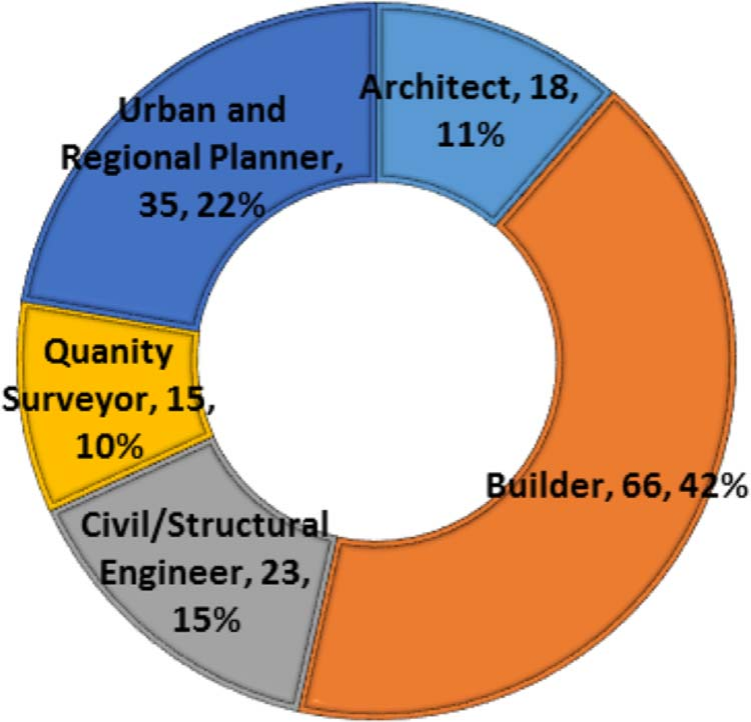
Breakdown of the participants.

**Fig. 2:**
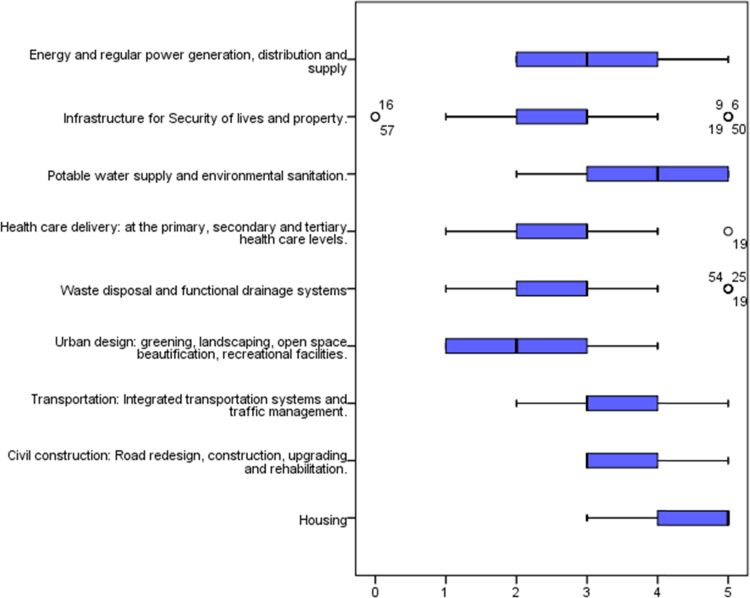
Boxplot of the state of housing and urban infrastructure.

**Fig. 3:**
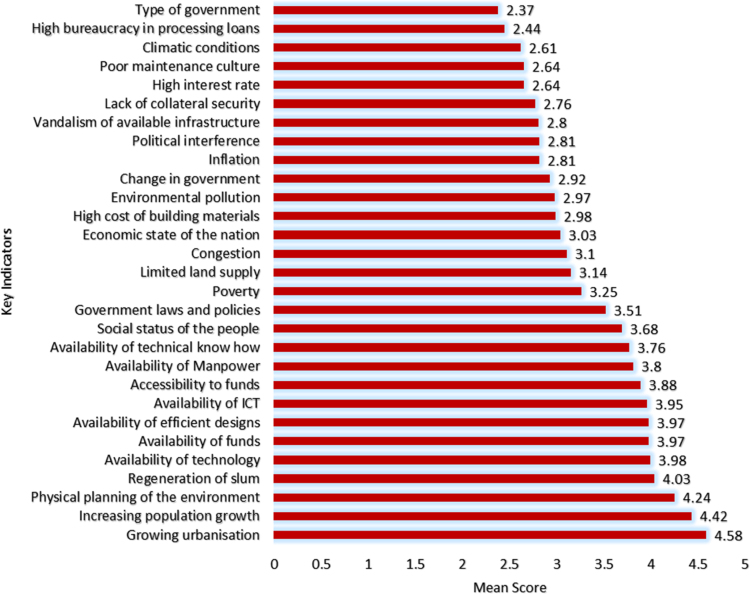
Key indicators spurring housing and urban infrastructures’ investment.

**Fig. 4:**
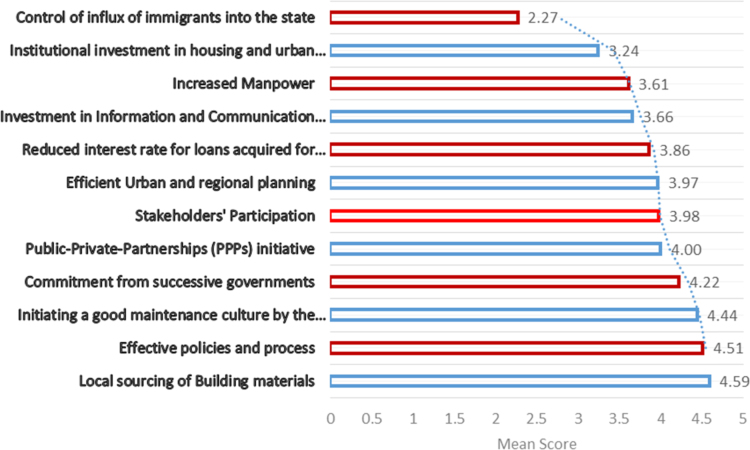
Improvement mechanisms to tackle housing and urban infrastructure deficit.

Continuous improvements are needed in cities that have been termed megacities due to the attendant challenges that arise from the increased population and uncontrollable rural-urban migration [1], [2], [3], [4]. One of such cities is Lagos state with over 20 million inhabitants in a landmass of 3577 km^2^ (approx. 0.4 percent of Nigeria's landmass). The unceasing developments needed in megacities in order to curtail the pressure on the environment and the citizens can be channeled through provision of adequate quantity and quality housing and urban infrastructure [5], [6], [7]. The statistical exploration of the dataset obtained contains state of housing and urban infrastructural from the perception of stakeholders, key indicators spurring the investment by government to meet the needs of the citizenry and improvement mechanisms that can be utilized to provide adequate housing and urban infrastructure for the teeming population of Lagos State. The data instrument utilized is a closed ended questionnaire which was measure on a 5-point Likert scale on selected variables from literature. The dataset consists of one hundred and fifty seven (157) responses from academics and construction professionals which included builders, architects, quantity surveyors, civil engineers, service engineers and urban and regional planners, all involved in the built environment and any other profession related to construction delivery services. The unique characteristics of these respondents are that they reside and work within the study area. Fig. 1 showed the breakdown of the participants involved in the dataset. In Table 1, the descriptive statistics presented the state of housing and urban infrastructures in the selected megacity. Key areas of housing and urban infrastructures which are abbreviated in Table 1 include: Housing: Provision of affordable and adequate housing (HO), Civil construction: Road redesign, construction, upgrading and rehabilitation (CC), Transportation: Integrated transportation systems and traffic management (TS), Urban design: greening, landscaping, open space beautification, recreational facilities (UD), Waste disposal and functional drainage systems to prevent flooding (WDS), Health care delivery: at the primary, secondary and tertiary health care levels (HCS), Potable water supply and environmental sanitation (PWS/ES), Security of lives and property (SI), and Energy and regular power generation, distribution and supply (ERPS). These abbreviations are further used in Table 2. Fig. 2 showed the variations using boxplot to depict the state of housing and urban infrastructure. Fig. 2 revealed that the dataset for the housing and urban infrastructure were not skewed. This depicts a normal distribution where the mean and the median are close, although, there are outliers experienced in WDS, HCD and SI. Furthermore, Fig. 3 displayed the key indicators spurring housing and urban infrastructures’ investment in megacities. The mean score of the key indicators were presented using stacked bars in Fig. 3. In order to measure the influence of some of the key indicators on the housing and urban infrastructures available in megacities, correlation matrix was used as shown in Table 2. Table 2 showed the correlation matrix key socio-economic indicators influencing housing and urban infrastructures’ investment. The selected socio-economic indicators include growing urbanization, increasing population growth, availability of funds, government laws and policies, change in government, availability of manpower, availability of technology, cost of building materials, economic state of the nation, inflation, environmental pollution and physical planning of the environment. In order to ensure a sustainable supply of adequate housing and urban infrastructure in megacities there is need for a carefully thought plan to fund and actualize a megacity master plan. The dataset showed some improvement mechanisms to tackle housing and urban infrastructure deficit as shown in Fig. 4. Inferential statistics using analysis of variance (ANOVA) helped measure if the measures were significant. Table 3 presented the analysis of variance (ANOVA) to measure significant measures to tackle housing and urban infrastructure deficit in megacities. There is need for megacities to periodically self-assess their supply of adequate housing and urban infrastructure for its populace so as to avert the propagation of slum, environmental pollution, congestion and aid urban planning. Policy makers and government officials need to be certain of areas to divert funds in terms of projects to undertake in megacities in the face of insufficient funds. This brings to light that the dataset would be useful in shedding light on measures that megacity officials can use in providing quantitative and qualitative supply of housing and urban facilities. The dataset can be replicated in order 42 megacities around the world on the state of housing and urban infrastructure available for its increasing population.

**Table 1:** Descriptive statistics on the state of housing and urban infrastructures.

	HO	CC	TS	UD	WDS	HCD	PWS/ES	SI	ERPS
Mean	4.64	3.61	3.12	2.17	2.95	2.76	3.83	2.76	3.37
Std. Error of Mean	.076	.091	.111	.135	.133	.088	.139	.135	.137
Median	5.00	3.00	3.00	2.00	3.00	3.00	4.00	3.00	3.00
Mode	5	3	3	1^a^	3	3	4	3	4
Std. Deviation	.580	.695	.853	1.036	1.024	.678	1.069	1.040	1.049
Variance	.337	.483	.727	1.074	1.049	.460	1.143	1.081	1.100
Skewness	− 1.415	.705	.457	.417	.603	.331	− .613	− .073	.024
Std. Error of Skewness	.311	.311	.311	.311	.311	.311	.311	.311	.311
Kurtosis	1.083	− .642	− .272	− .982	− .114	1.383	− .820	1.034	− 1.213
Std. Error of Kurtosis	.613	.613	.613	.613	.613	.613	.613	.613	.613
Total Respondents	157	157	157	157	157	157	157	157	157

a Multiple modes exist. The smallest value is shown.

**Table 2:** Correlation matrix of key socio-economic indicators influencing housing and urban infrastructures' investment.

**Key indicators**		**HO**	**CC**	**TS**	**UD.**	**WDS**	**HCD**	**PWS/ES**	**SI**	**ERPS**
Growing urbanization	Pearson Correlation	−.047	−.165	−.109	−.229	−.038	−.177	.365**	−.116	.068
	Sig. (2− tailed)	.722	.212	.412	.081	.775	.179	.004	.383	.610
	N	157	157	157	157	157	157	157	157	157
Increasing population growth	Pearson Correlation	.106	−.221	−.123	−.291*	.047	−.065	.223	−.266*	−.350**
	Sig. (2-tailed)	.425	.092	.352	.025	.724	.627	.090	.041	.007
	N	157	157	157	157	157	157	157	157	157
Availability of funds	Pearson Correlation	.146	−.222	−.041	−.376**	−.524**	−.423**	.235	.182	.090
	Sig. (2-tailed)	.268	.092	.758	.003	.000	.001	.074	.169	.500
	N	157	157	157	157	157	157	157	157	157
Government laws and policies	Pearson Correlation	.019	.134	−.030	−.206	−.008	−.166	−.060	.080	.028
	Sig. (2-tailed)	.886	.313	.822	.117	.955	.210	.653	.545	.832
	N	157	157	157	157	157	157	157	157	157
Change in government	Pearson Correlation	−.021	.320*	.402**	.426**	.460**	.229	−.250	.317*	.058
	Sig. (2-tailed)	.873	.013	.002	.001	.000	.080	.056	.014	.660
	N	157	157	157	157	157	157	157	157	157
Availability of Manpower	Pearson Correlation	−.276*	−.402**	−.428**	−.530**	−.357**	−.125	.082	.215	−.536**
	Sig. (2-tailed)	.034	.002	.001	.000	.005	.346	.539	.102	.000
	N	157	157	157	157	157	157	157	157	157
Availability of technology	Pearson Correlation	−.226	.026	.181	−.228	.057	−.366**	−.371**	.283*	−.334**
	Sig. (2-tailed)	.085	.842	.170	.083	.666	.004	.004	.030	.010
	N	157	157	157	157	157	157	157	157	157
Cost of building materials	Pearson Correlation	−.276*	−.010	.183	.135	.199	.070	−.514**	.062	−.075
	Sig. (2-tailed)	.035	.943	.166	.309	.130	.600	.000	.641	.570
	N	157	157	157	157	157	157	157	157	157
Economic state of the nation	Pearson Correlation	−.217	−.030	.359**	.244	.120	−.039	−.543**	.290*	−.177
	Sig. (2-tailed)	.099	.820	.005	.063	.367	.770	.000	.026	.180
	N	157	157	157	157	157	157	157	157	157
Inflation	Pearson Correlation	−.272*	−.007	.339**	.254	.302*	.271*	−.346**	.109	.069
	Sig. (2-tailed)	.037	.956	.009	.052	.020	.038	.007	.412	.602
	N	157	157	157	157	157	157	157	157	157
Environmental pollution	Pearson Correlation	−.313*	.054	.064	.022	.296*	.138	−.339**	−.334**	−.586**
	Sig. (2-tailed)	.016	.684	.628	.869	.023	.297	.009	.010	.000
	N	157	157	157	157	157	157	157	157	157
Physical planning of the environment	Pearson Correlation	.212	−.016	−.088	−.305*	.034	−.051	.084	−.509**	−.338**
	Sig. (2-tailed)	.106	.904	.506	.019	.796	.702	.526	.000	.009
	N	157	157	157	157	157	157	157	157	157

* Correlation is significant at the 0.05 level (2-tailed).

** Correlation is significant at the 0.01 level (2-tailed).

**Table 3:** ANOVA to measure significant measures to tackle housing and urban infrastructure deficit.

**Measures**		**Sum of Squares**	**df**	**Mean Square**	**F**	**Sig.**
Effective policies and process	Between Groups	2.000	4	.500	1.441	.233
	Within Groups	18.745	153	.347		
	Total	20.746	157			
Efficient Urban and regional planning	Between Groups	4.505	4	1.126	1.717	.160
	Within Groups	35.427	153	.656		
	Total	39.932	157			
Local sourcing of Building materials	Between Groups	2.267	4	.567	1.532	.206
	Within Groups	19.970	153	.370		
	Total	22.237	157			
Institutional investment in housing and urban development projects	Between Groups	18.683	4	4.671	3.603	.011
	Within Groups	69.995	153	1.296		
	Total	88.678	157			
Public-Private-Partnerships (PPPs) initiative	Between Groups	8.223	4	2.056	3.728	.009
	Within Groups	29.777	153	.551		
	Total	38.000	157			
Stakeholders' Participation	Between Groups	4.456	4	1.114	1.970	.112
	Within Groups	30.527	153	.565		
	Total	34.983	157			
Initiating a good maintenance culture by the government and the public	Between Groups	1.531	4	.383	.984	.424
	Within Groups	21.011	153	.389		
	Total	22.542	157			
Increased Manpower	Between Groups	5.125	4	1.281	1.982	.110
	Within Groups	34.909	153	.646		
	Total	40.034	157			
Investment in Information and Communication Technology (ICT)	Between Groups	3.725	4	.931	.977	.428
	Within Groups	51.495	153	.954		
	Total	55.220	157			
Reduced interest rate for loans acquired for infrastructural projects	Between Groups	7.445	4	1.861	2.833	.033
	Within Groups	35.470	153	.657		
	Total	42.915	157			
Control of influx of immigrants into the state	Between Groups	14.341	4	3.585	1.650	.175
	Within Groups	117.320	153	2.173		
	Total	131.661	157			
Commitment from successive governments	Between Groups	2.402	4	.600	1.090	.371
	Within Groups	29.734	153	.551		
	Total	32.136	157			

## 2 Experimental design, materials and methods

The dataset was collected in a major commercial city in Nigeria, in Lagos State. Lagos is the commercial capital of Nigeria and spatially the smallest state in the country an area approximately 3577 sq. km, out of which 39% are wetlands. Other dataset that have been carried out within this region can be found in [8], [9], [10], [11]. Presently, Lagos State is regarded as the most populous city in sub-Saharan Africa, with a population of over 20 million people ranking it the sixth megacity in the world. The population of which the dataset was obtained consist of construction professionals involved in the built environment. A convenience sampling technique was used in selecting one hundred and fifty seven (157) professionals for the dataset due to the characteristics of the respondents and the specifics of the dataset. The questionnaire instrument had four (4) sections which include the background information of the participants, the state of housing and urban infrastructure, the key indicators influencing housing and urban infrastructure investment and the measures to actualize the adequate provision of housing and urban infrastructure. Descriptive statics was used to present each section inform of tables, pie chart and stacked bars. Inferential statistics were formulated to test the influence of key indicators on the housing and urban infrastructure parameters. In addition, the significant difference of the improvement mechanisms for adequate provision of housing and urban infrastructures in megacities. Descriptive and inferential statistics as used in other works can be found in [12], [13], [14], [15], [16], [17], [18]. The problems posed by growing urbanization in megacities should be adequately tackled in order to prevent the development of residential slums. Inability to match the housing needs with available resources to accommodate the population explosion would impinge negatively on available urban infrastructure. Future dataset should be focused on giving the actual figures of the deficit that exist in the provision of housing and urban infrastructure and the public-private participation in a sustainable housing and urban development model within megacities. The key indicators can be explored individually to examine the micro-effect on available facilities in megacities.
