# Supplementary material for: Statistical exploration of dataset examining key indicators influencing housing and urban infrastructure investments in megacities
Source: Data Brief. 2018 May 2;18:1725–33. doi: 10.1016/j.dib.2018.04.089 (PMC5998746; doi:10.1016/j.dib.2018.04.089)
Supplement: Supplementary file 1 — Transparency document [file mmc1.docx]

**COVENANT UNIVERSITY**

**COLLEGE OF SCIENCE AND TECHNOLOGY**

CANAANLAND, KM 10, IDIROKO ROAD

P.M.B 1023, OTA, OGUN STATE, NIGERIA

www.covenantuniversity.edu.ng

EXTERNAL MEMO

**To:** Editor, Data in Brief

**From:** Corresponding Author

**Date:** 7^th^ March, 2018

**Subject: Conflict of Interest**

The above subject refers. The authors of the manuscript hereby declare the absence of any conflict of interest among the authors in the submission to Data in Brief. The authors have read the final draft and unanimously agreed that the paper be sent for review.

Regards,

Adedeji O. Afolabi

Department of Building Technology,

Covenant University, Ota.

adedeji.afolabi@covenantuniversity.edu.ng
